# Multigenic Control of Pod Shattering Resistance in Chinese Rapeseed Germplasm Revealed by Genome-Wide Association and Linkage Analyses

**DOI:** 10.3389/fpls.2016.01058

**Published:** 2016-07-21

**Authors:** Jia Liu, Jun Wang, Hui Wang, Wenxiang Wang, Rijin Zhou, Desheng Mei, Hongtao Cheng, Juan Yang, Harsh Raman, Qiong Hu

**Affiliations:** ^1^Key Laboratory of Biology and Genetic Improvement of Oil Crops, Ministry of Agriculture, Oil Crops Research Institute of the Chinese Academy of Agricultural SciencesWuhan, China; ^2^Graduate School of Chinese Academy of Agricultural SciencesBeijing, China; ^3^Graham Centre for Agricultural Innovation (an Alliance between NSW Department of Primary Industries and Charles Sturt University), Wagga Wagga Agricultural InstituteWagga Wagga, NSW, Australia

**Keywords:** rapeseed, pod shatter resistance, genetic linkage mapping, genome-wide association, design breeding

## Abstract

The majority of rapeseed cultivars shatter seeds upon maturity especially under hot-dry and windy conditions, reducing yield and gross margin return to growers. Here, we identified quantitative trait loci (QTL) for resistance to pod shatter in an unstructured diverse panel of 143 rapeseed accessions, and two structured populations derived from bi-parental doubled haploid (DH) and inter-mated (IF_2_) crosses derived from R1 (resistant to pod shattering) and R2 (prone to pod shattering) accessions. Genome-wide association analysis identified six significant QTL for resistance to pod shatter located on chromosomes A01, A06, A07, A09, C02, and C05. Two of the QTL, *qSRI.A09* delimited with the SNP marker Bn-A09-p30171993 (A09) and *qSRI.A06* delimited with the SNP marker Bn-A06-p115948 (A06) could be repeatedly detected across environments in a diversity panel, DH and IF_2_ populations, suggesting that at least two loci on chromosomes A06 and A09 were the main contributors to pod shatter resistance in Chinese germplasm. Significant SNP markers identified in this study especially those that appeared repeatedly across environments provide a cost-effective and an efficient method for introgression and pyramiding of favorable alleles for pod shatter resistance via marker-assisted selection in rapeseed improvement programs.

## Introduction

Rapeseed (*Brassica napus* L., 2n = 4× = 38, genome AACC) is the third largest oilseed crop produced in the world after oil palm and soybean (USDA FAS, 2015)^1^. In nature, many plant species including rapeseed dehisce seeds easily upon maturity for dispersal and survival in subsequent generations. However, this phenomenon is one of the major bottlenecks in rapeseed production on a commercial scale. The yield loss due to seed shatter usually accounts for about 5–10% of total production; and under relatively harsh climatic conditions, it can reach up to 50% (Kadkol et al., 1984; Price et al., 1996). Moreover, shattered seeds become “volunteers” in subsequent crops in the rotation cycle, making crop management difficult and expensive (Morgan et al., 2000). Rapeseed is generally harvested by windrowing or swathing. However, in recent years, farmers prefer to use combine harvesters, as this operation is less-labor intensive and cheaper compared to windrowing and manual harvesting. The latter is not an option for many western countries where rapeseed is often used as a broad-acre crop and harvested under very hot and dry conditions. Therefore, developing pod shatter resistant varieties suitable for combine harvesting has become one of the main breeding objectives of rapeseed improvement programs.

A limited genetic variation exists for pod shatter resistance in natural germplasm of rapeseed (Morgan et al., 1998; Wen et al., 2008). For example, Wen et al. (2008) evaluated 229 genotypes of rapeseed and identified only two genotypes having moderate levels of resistance to pod shatter. However, genetic variation for higher levels of resistance to pod shatter is present in other close relatives of rapeseed, such as *Brassica rapa, Brassica juncea*, and *Brassica carinata* (Kadkol et al., 1984; Mongkolporn et al., 2003; Raman et al., 2014). These related species have been utilized to improve pod shatter resistance in rapeseed via interspecific hybridization (Liu, 1994; Wei et al., 2010; Raman et al., 2014).

To gain insight into the genetic basis underlying quantitative variation in traits of agricultural significance such as pod shatter resistance and to enhance predictive selection efficiency in plant breeding programs, genetic mapping has become an important tool (Mauricio, 2001). Recent developments in next-generation sequencing technology, discovery of high throughput marker systems such as high density SNP markers (Trick et al., 2009; Bancroft et al., 2011), genotyping-by-sequencing (Raman et al., 2014; Bayer et al., 2015) and sequence capture (Schiessl et al., 2014), availability of chromosome based sequence of *B. rapa, B. oleracea, and B. napus* genomes (Wang et al., 2011; Chalhoub et al., 2014; Liu et al., 2014; Parkin et al., 2014) and bioinformatics, have enabled improving genomic selection of desirable alleles through marker-assisted selection in rapeseed. Multigenic inheritance for pod shatter resistance has been reported in *B. rapa*, and *B. napus* (Kadkol et al., 1986; Hossain et al., 2011; Wen et al., 2013). During the last 5 years, up to 10 QTL associated with resistance to pod shatter have been identified in both genetic mapping populations derived from doubled haploid (DH) lines (Hu et al., 2012; Wen et al., 2013; Raman et al., 2014) and a diversity panel of rapeseed accessions, originated mainly from Australia (Raman et al., 2014). Genetic loci associated with pod shatter resistance has also been mapped in *B. rapa* using RAPD markers (Mongkolporn et al., 2003), and soybean (Gao and Zhu, 2013). Several genes such as *IND, ALC, SHP1, SHP2*, and *FUL* and their complex regulatory network involved in pod dehiscence have been identified in *Arabidopsis*, rice and soybean (Ferrándiz et al., 2000; Liljegren et al., 2000; Rajani and Sundaresan, 2001; Konishi et al., 2006; Lewis et al., 2006; Li et al., 2006; Østergaard, 2009; Zhou et al., 2012; Dong et al., 2014; Funatsuki et al., 2014; Yoon et al., 2014).

In this study, we performed a genome wide association study (GWAS) in a diversity panel of 143 accessions and classical QTL analyses utilizing a DH population and inter-mated F_2_ (IF_2_) population derived from R1 (resistant to pod shatter) and R2 (prone to pod shatter) rapeseed advanced breeding lines of Chinese origin to identify loci involved in pod shatter resistance. The publicly available 60K Brassica Infinium® SNP array was utilized to genotype mapping populations. We uncovered that pod shatter resistance is controlled by multiple loci having both major and minor allelic effects. Identification of loci via GWAS and classical QTL analyses, and SNP marker significantly associated with pod shatter resistance may facilitate a cost-effective marker assisted selection of favorable alleles in rapeseed breeding programs.

## Materials and methods

### Association mapping population

A total of 143 diverse rapeseed accessions including 6 elite winter types, 124 semi-winter types, and 13 spring types were used for GWAS (Supplementary Table 1). Based on their origins, 112 accessions originated from China, 24 from Oceania, 5 from Europe, 1 from North America, and 1 from India. This GWAS panel also included parental lines; R1 and R2 utilized for the development of DH and IF_2_ populations investigated in this study. The seeds of all accessions were procured from the National Mid-term Genebank for Oil Crops, Wuhan, China, and then multiplied at the Oil Crops Research Institute of the Chinese Academy of Agricultural Sciences (OCRI-CAAS), Wuhan, China. All accessions were planted in a field following a randomized complete block design with 2 replications in 3 consecutive years (2011, 2012, and 2013) at Yangluo Research Station (248 310S; 338 00E) in Hubei, China. Seeds were sown at normal agronomic density in plots of 2 × 1 m. Each plot contained three rows; each row with 18 plants. Field management followed the standard agricultural practice.

### DH genetic mapping population

A mapping population, designated as RR, comprising 96 DH lines was developed from an F_1_ plant derived from the cross of R1 (maternal parent) and R2 (paternal parent). The R1 and R2 were elite semi-winter breeding lines developed by OCRI-CAAS. R1 is a highly resistant advanced breeding line to pod shatter (Liu J. et al., 2013) whereas R2 is a highly prone to pod shattering line under field conditions; both lines are paternal lines of two high yielding commercial hybrid cultivar in China. The RR-DH population was grown in consecutive 2 years, i.e., 2013 and 2014 under winter-cropped environments at Yangluo Research Station and phenotyped for pod shatter resistance.

### Construction of immortalized F_2_ (IF_2_) validation population

In order to verify the genetic associations between SNP markers and pod shatter resistance identified in a RR-DH population and to understand additive interaction among loci, all DH lines were intercrossed following a random permutation design (Hua et al., 2002) for constructing an immortalized F_2_ (IF_2_) population. The random permutation was repeated three times. In each permutation, the 96 DHs were randomly divided into two groups, and the 48 lines in each group were paired up at random to a counterpart in the other group by taking one line from each group for one cross at a time and taking one from the rest lines for the next cross to ensure that each DH line was used only once in each round of permutation. Pairs with the same two parental lines from the three repeated permutation were manually corrected to eliminate identical pairings. In theory, 48 IF_2_ crosses should be produced from each round and in total 144 crosses could be obtained from the three repeats. However, some combinations failed to obtain seeds due to an asynchronous flowering of the parental DH lines, resulting in a total of 124 IF_2_ derivatives. All parental DH lines and their hybrid derivatives (F_1_) were planted in a randomized complete block design in Yangluo Experimental Station in 2013 winter season. Seeds were sown at normal agronomic density in plots (2 × 1 m/plot). Each plot contained three rows with 18 plants in each row. Field management followed the standard agricultural practice.

### Assessment for resistance to pod shattering

At physiological maturity, 10 plants from the middle of the plots were harvested to evaluate their resistance to pod shatter. Ten pods from each plant were taken from the main inflorescence and then bulked to make a composite sample for measuring pod shatter resistance index (PSRI) using a modified random impact test (RIT; Peng et al., 2013). Samples of mature pods were first oven dried at 45°C for 8 h and then subjected to shaking at 300 rpm in a drum with an inner diameter of 20 cm and a height of 12 cm, together with ball bearings (14 mm diameter). In this laboratory-based RIT procedure, the number of dispersed pods was recorded five times at 2 min intervals of standardized shaking. The PSRI was calculated as follows: PSRI = 1− $\sum_{i=5}^{i=1}xi\times (6-i)$ /100, where *x*_*i*_ is the number of ruptured pods at the *i*th time (1 ≤ *i* ≤ 5).

### SNP genotyping

Genomic DNA was isolated from pooled samples of young leaves from 5 plants of each genotype using a CTAB method (Saghai-Maroof et al., 1984). DNA content of each sample was measured using Nanodrop spectrometer (Model ND-2000). The DNA samples were genotyped with the Illumina Brassica 60K Infinium® SNP array as per manufacture's protocol (Illumina Inc., San Diego, USA) by Emei Tongde Co. (Beijing). The SNP data were clustered and called using the Genome Studio genotyping software (Illumina). Among the three possible genotypes (AA, AB, and BB), genotypes with AB alleles was excluded, the remaining homozygous SNP markers were selected to carry out genetic analyses. Genotypic data were curated to remove those SNPs with AA or BB frequency equal to zero, call rates ≥0.8 and minor allele frequency <0.05.

### Construction of a high density SNP genetic map

The software IciMapping V4.0 (Wang et al., 2014, http://www.isbreeding.net/software/?type=detail&id=14) was used to “bin” redundant markers with exactly the same genotypes. Distortion in segregating SNP markers was checked using the χ^2^ test according to the expected segregation ratio [AA(1): BB(1)] in DH population. Non-redundant SNP markers showing 1:1 segregation ratio were then used for construction of the genetic linkage map using the software JoinMap version 4.0 (Stam, 1993, https://www.kyazma.nl/index.php/mc.JoinMap), using a recombination frequency of < 0.25 and minimum LOD score of 5. Recombination frequencies were converted using Kosambi's algorithm (Kosambi, 1944). Linkage groups were assigned to chromosomes A01 to A10 and C01 to C09 according to published genetic maps (Liu L. et al., 2013; Brown et al., 2014; Wang et al., 2015).

### *In silico* mapping of SNP markers

In order to verify the chromosomal location of SNP markers and to compare their physical positions in relation to the known genes involved in pod shatter resistance in *Arabidopsis thaliana* and *B. napus* (www.tair.com, Girin et al., 2010; Hu et al., 2012; Raman et al., 2014; Dong and Wang, 2015), sequences of all associated SNPs and candidate genes were used to perform BlastN searches against the *B. napus* cv. Darmor genome sequence (Chalhoub et al., 2014). Only the top blast-hits with an *E*-value cut-off of 1E^−15^ were considered for genetic and comparative analyses. The closest known pod shatter resistance gene in relation to the physical position of SNP marker on the *B. napus* genome was assumed to be a “candidate” gene for pod shatter resistance in genetic mapping populations.

### Statistical analysis and QTL identification

The PROC GLM procedure was used to estimate the variance components for individual traits/environments using SAS software version 8.1 (SAS Institute Inc., 1999). Genotype was considered a fixed effect, whereas environment was considered as random effects. The mean value of the trait was calculated and then used for genetic analysis.

The model of composite interval mapping (CIM) in the WinQTL cartographer version 2.5 (Wang et al., 2007) was used for QTL identification. Multiple linear regression was conducted using forward-backward stepwise and a probability model was set with 0.05 and window size at 10 cM. The LOD threshold was determined by 1000 permutation test (Churchill and Diverge, 1994) and a significant level of 0.01 were selected to determine whether there is any QTL for pod shatter resistance.

### Population structure, kinship, and GWA analysis

For GWAS, three data types are required: genotypic data, population structure within the GWA panel (population) and phenotypic trait information. After discarding SNP markers which were either monomorphic and/or had minor allele frequencies (MAFs) <0.05, a total of 66.1% (34,469/52,157) high-quality polymorphic SNPs were selected for GWAS.

In order to infer the population structure of the GWAS panel, a subset of data of 2434 SNP markers which showed genome-wide coverage across all 19 chromosomes were used into the software package STRUCTURE version 2.3.4 (Pritchard et al., 2000). An admixture model was performed for five independent runs with a *K*-value, ranging from 1 to 10, iterations of 100,000 times, burn-in period of 100,000 MCMC (Markov Chain Monte Carlo). The optimal *K*-value was determined according to the method of Evanno et al. (2005). The cluster membership coefficient matrices of replicate runs from STRUCTURE were integrated to get a Q matrix by the CLUMPP software (Jakobsson and Rosenberg, 2007). Accessions with the probability of membership >0.7 were assigned to corresponding clusters, and those < 0.7 were assigned to a mixed group. Q matrices were used as covariates to calculate population structure with K. The extent of LD for each chromosome was estimated using pairwise *r*^2^ of all mapped SNPs using window of 500.

With Best linear unbiased predictors (BLUPs) of calculated for all phenotypic environments (3 years, Table 1), we conducted a GWAS with 34,469 genome-wide SNPs using a univariate unified mixed linear model (Yu et al., 2006) that eliminated the need to recomputed variance components (i.e., population parameters previously determined, or P3D; Zhang et al., 2010). To control the effect of familial relatedness in GWAS, the kinship matrix based on coancestry (Loiselle et al., 1995) was estimated using 34,469 genome-wide SNPs. A likelihood-ratio-based *R*^2^ statistic, denoted *R*^2^ LR (Sun et al., 2010), was used to assess the amount of phenotypic variation explained by the model. The Benjamini and Hochberg (1995) procedure was used to control the multiple testing problem at false-discovery rates (FDRs) of 5 and 10%. GWAS was performed by TASSEL 4.0 (Bradbury et al., 2007) using a mixed linear model (MLM) in which relative kinship matrix (K) and population structure (Q) were included as fixed and random effects, respectively. Significance of associations between traits and SNPs was set on threshold *P* < 2.90 × 10^−5^ (i.e., −log_10_(*p*) = 4.5). The threshold is 2.90 × 10^−5^ at a significant level of 1% after Bonferroni multiple test correction (1/34,496). Furthermore, the false discovery rate (FDR at *P* < 0.05) was applied to estimate the proportion of false positives among the significant associations (Dabney and Storey, 2004). The marker effect and the significant value generated in R package for each SNP were exported (http://cran.r-project.org). LD block analysis was performed as described previously, keeping the lead SNP within each LD block (Gabriel et al., 2002).

**Table 1:** **Genetic variation and broad-sense heritability in pod shatter resistance index (PSRI) among three populations**.

**Population**	**Phenotyping environment (year)**	**PSRI (range)**	**PSRI (Mean ± SD)**	**CV (%)**	**Genotype (G)**	**Environment (E)**	**G × E**	**H^2^(%)**
DH	2013	0.01–0.58	0.14 ± 0.12	92.49	^**^	^**^	^**^	85.11
	2014	0.03–0.99	0.46 ± 0.28	61.48				
IF^2^	2014	0.05–0.99	0.50 ± 0.27	53.70	^**^			
GWAS diversity set	2011	0.00–0.58	0.09 ± 0.11	119.32	^**^			92.11
	2012	0.01–0.64	0.15 ± 0.14	87.54				
	2013	0.00–0.71	0.09 ± 0.14	154.04				

** *P < 0.01 for the effect of genotype (G), environment (E), and genotype by environment interaction (G × E) on phenotypic variance estimated by two-way ANOVA*.

*CV, coefficient of variation; H^2^, broad sense heritability; SD, Standard deviation*.

### Allelic effects of pod shatter accessions

Based on pod shatter resistance indices, all 143 accessions were ranked and then investigated for allelic diversity at significant GWAS SNP loci. PSRI of R1 and R2 were 0.45 and 0.04, respectively. Accessions having PSRI ≥ 0.28 were assumed to have superior alleles for pod shatter resistance.

## Result

### Genetic variation for pod shatter resistance in biparental populations

**Figure 1:**
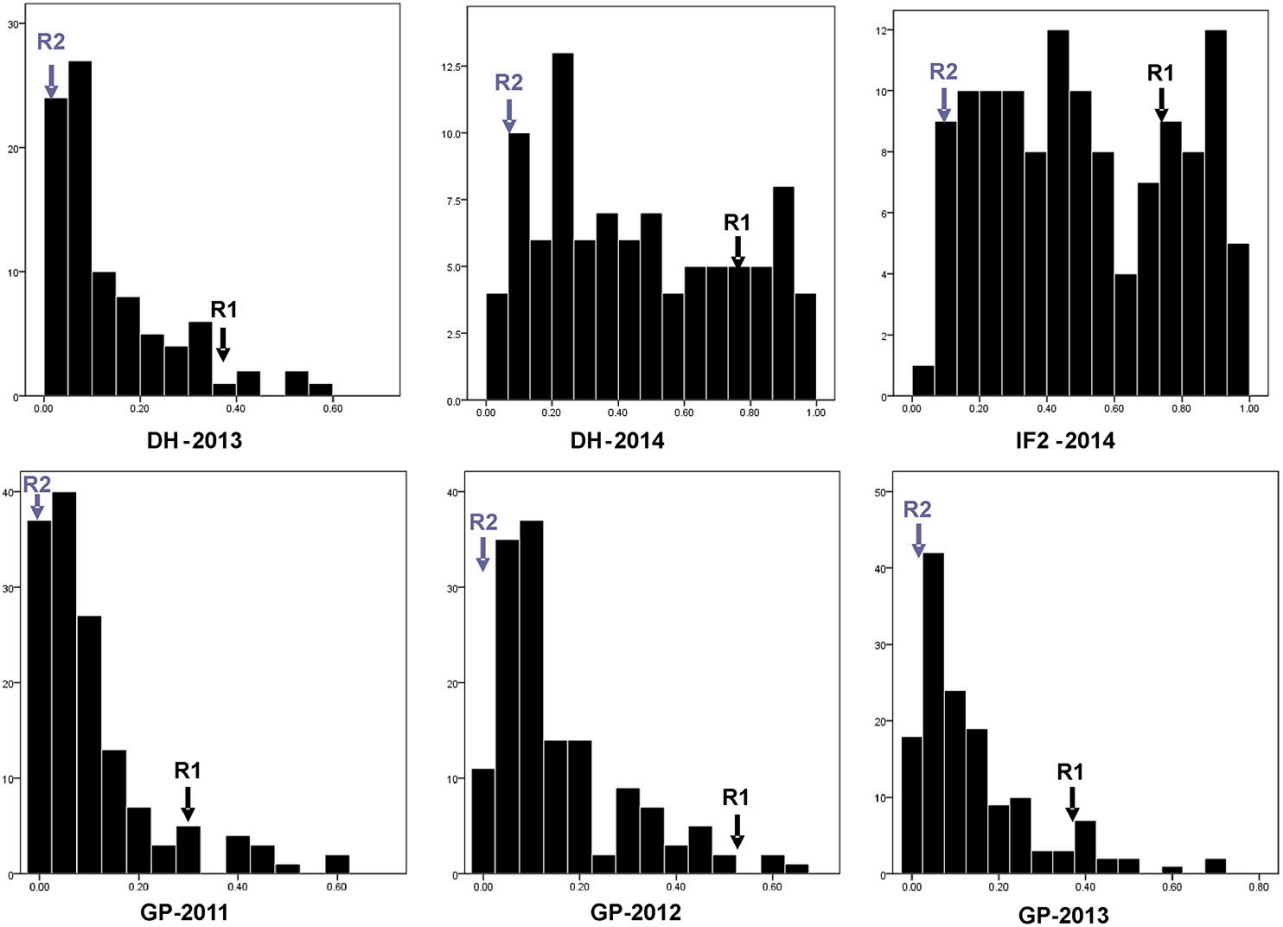
**Phenotypic distribution for individual pod shatter resistance index in DH population (R1 × R2) across 2 years (2013 and 2014) and IF_**2**_ population (2014), and GWAS panel (GP) across 3 years (2011, 2012, and 2013)**. Transgressive segregation was observed in the DH and IF_2_ populations from all of the environments. Gray arrows are for R2 and black arrows are for R1. y axis represents Number of lines and accessions and x axis represents pod shatter resistance index measured by RIT (Random Impact Test) method.

**Figure 2:**
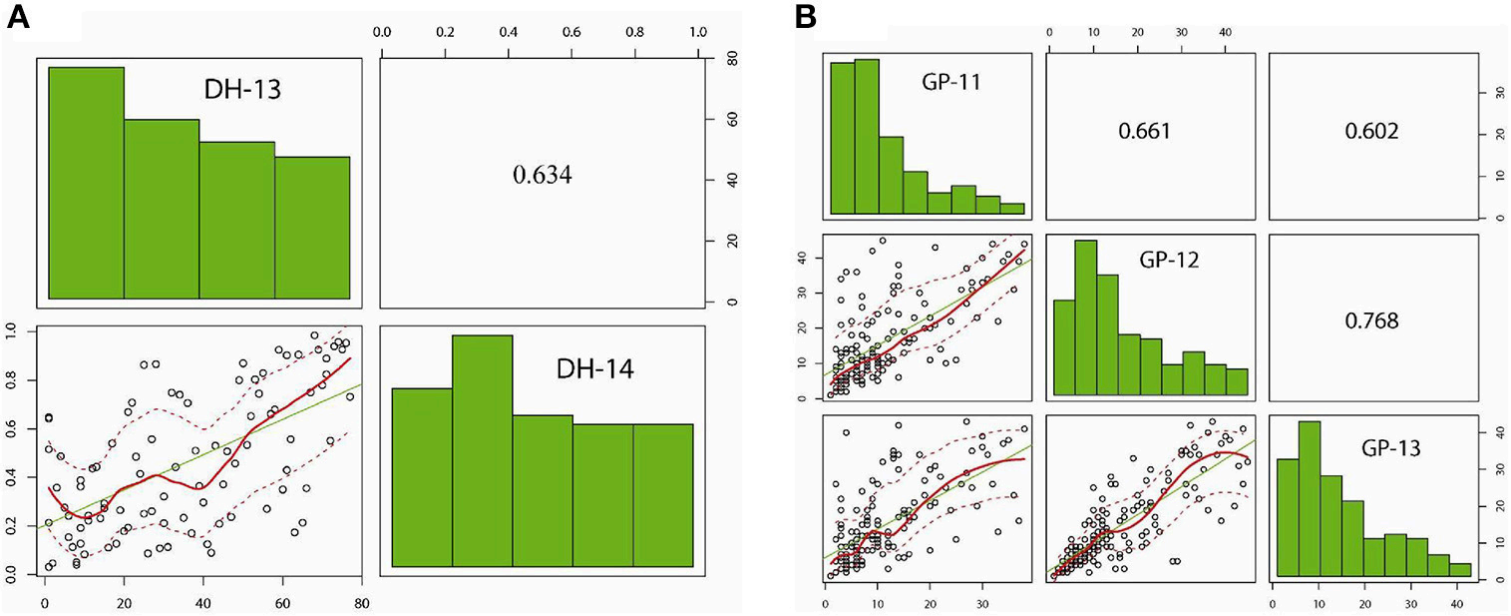
**Distribution of pod shatter resistance, as measured with the random impact test, among DH lines from the R1/R2 and GWAS diversity set**. Pair-plots of EBLUPS from DH lines and GWAS diversity set showing correlations are presented. **(A)** R1/R2 population grown under two environments: experiment 1 (DH-13); experiment 2 (DH-14). **(B)** GWAS diversity set grown under three environments: GP-11, GP-12, and GP-13.

Predicted means for PSRI of DH and IF_2_ populations showed a continuous distribution for pod shatter resistance irrespective of growing environments. Both parental lines differed significantly in pod shatter resistance across all phenotyping environments. R1, the resistant parent, had consistently higher PSRI (0.45) compared to the pod shatter prone parent, R2 (0.04; Figure 1). The frequency distribution of PSRI deviated significantly from normality among DH and IF_2_ lines (*P* < 0.001). Among RR-DH lines, a strong positive correlation (*r* = 0.60) of genotype performance for PSRI was observed across 2013 and 2014 environments (Figure 2), suggesting that phenotypic variation in PSRI is genetically controlled, consistent with high broad-sense heritability values (Table 1). Analysis of variance showed that the effects of genotype (G), and genotype × environment (G × E) interaction on PSRI were significant (Table 1), suggesting that genetic mapping populations must be evaluated across multiple sites/years to ensure valid phenotypic assessment.

### Construction of a high-density genetic bin map for QTL analysis

**Figure 3:**
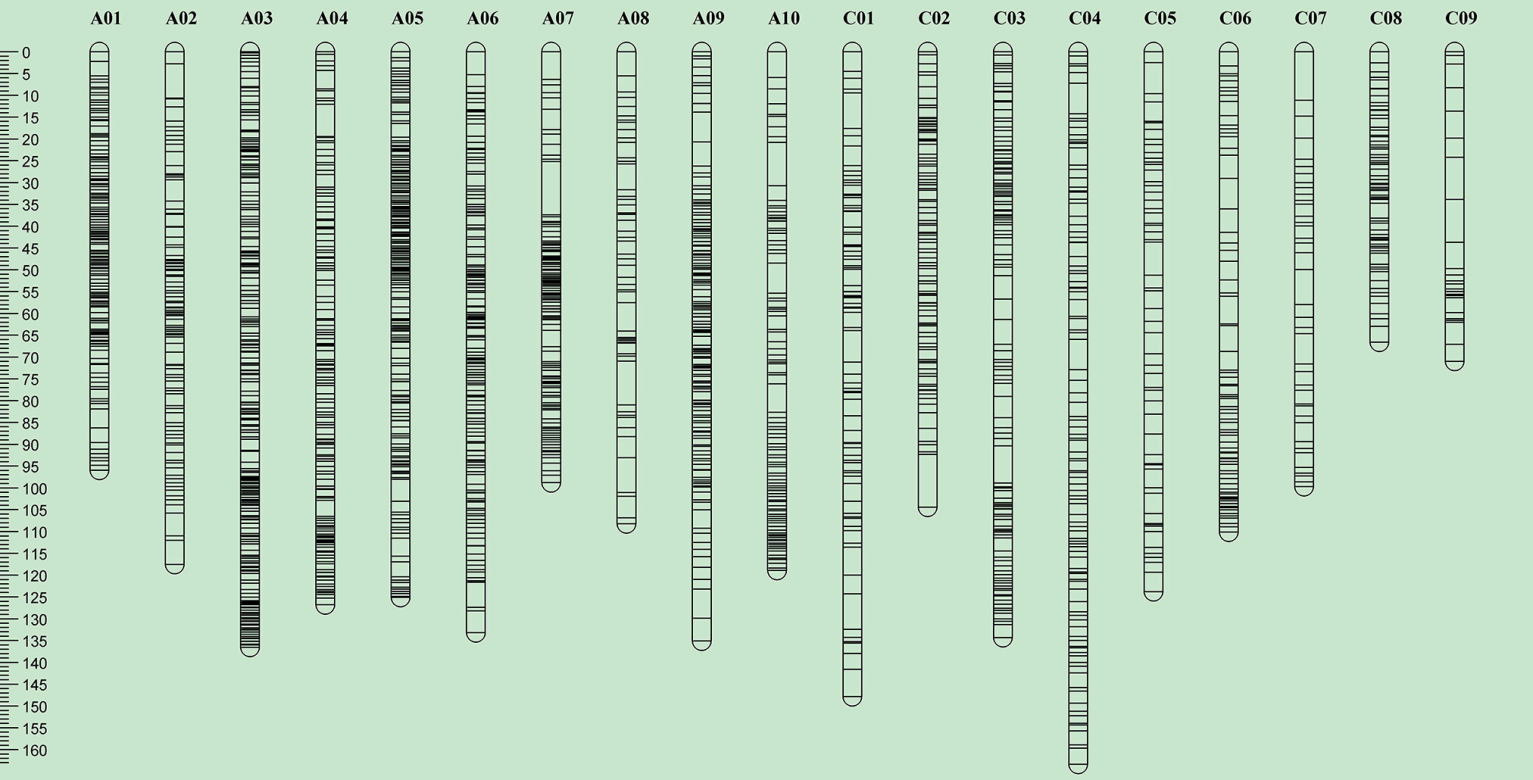
**Overview of genome-wide SNP density in the bin map of the RR-DH population derived from R1 (resistant to pod shatter) and R2 (prone to pod shatter) lines of ***B. napus*****. The ordinate shows the genetic distance along each of the 19 linkage groups corresponding to the 19 *B. napus* chromosomes. Scale in Kosambi centimorgans (cM) is on the left.

Of the 52,157 SNP markers (60K Infinium array), only 16.4% (8540) were polymorphic between the parental lines, R1 and R2 of the RR-DH population. Of these, 7804 SNP markers showing 1:1 segregation ratio, as determined by the χ^2^ test (*P* = 0.05), were used for construction of a genetic linkage map and QTL analysis. A majority (99%) of the polymorphic markers (7728/7804) were anchored to the 19 chromosomes of *B. napus* and mapped to 2046 distinct loci, with 1384 loci on A genome, and 662 loci on the C genome (Table 2, Figure 3). A total of 5682 SNP loci showed co-segregation and could be grouped into 900 discrete bins. A genetic linkage map of RR-DH population spanned 2217.2 cM of Kosambi map distance. The marker density of the 19 chromosomes ranged from 0.61 (A03) to 2.96 (C09), with an average of 1.08 cM. The chromosome A03 displayed the maximum marker density (738 markers representing 222 loci) and chromosome C09 had the least density (77 markers representing 24 loci). In particular, chromosomes C08 and C09 were shorter (66.6–71 cM) than rest of the chromosomes (Table 2).

**Table 2:** Features of the genetic linkage map of a DH population derived from R1 /R2 of B. napus^*^.

**Chromosome**	**No. of polymorphic markers**	**No. of mapped markers**	**No. of mapped markers in bin loci**	**No. of bin loci**	**Map length (cM)**	**Average distance between loci (cM)**
A01	329	233	145	49	95.9	0.66
A02	317	269	98	50	117.5	1.20
A03	738	612	222	96	136.5	0.61
A04	191	448	139	60	126.8	0.91
A05	615	498	199	84	125.1	0.63
A06	450	359	158	67	133.2	0.84
A07	483	417	122	56	98.8	0.81
A08	269	237	52	20	108.2	2.08
A09	632	546	147	61	135.1	0.92
A10	191	115	102	26	118.9	1.17
C01	405	361	81	37	147.9	1.83
C02	891	852	94	55	104.4	1.11
C03	665	602	123	60	134.3	1.09
C04	574	526	106	58	163.4	1.54
C05	147	116	56	25	123.8	2.21
C06	500	469	73	42	110.1	1.51
C07	794	769	39	14	99.7	2.56
C08	272	239	66	33	66.6	1.01
C09	77	60	24	7	71.0	2.96
Subtotal for the A genome	4215	3734	1384	569	1196.0	0.86
Subtotal for the C genome	4325	3994	662	331	1021.2	1.54
Total (A+C)	8540	7728	2046	900	2217.2	1.08

* *Markers which showed co-segregation with each other were binned using the ICI mapping package (http://www.isbreeding.net/software/?type=detail&id=14)*.

The SNP genotypes of 124 F_1_ hybrids were deduced from their corresponding DH parental lines to provide a bin map for the IF_2_ crosses (Figure 3). There were three genotypes in each bin: homozygous genotype from R1 (*MM*), homozygous genotype from R2 (*mm*), and heterozygous genotype (*Mm*). The average proportion of three genotypes for each cross was 27.3, 29.2, and 43.5%, respectively. Therefore, the composition of genotypes in IF_2_ was similar to that in an F_2_ population. This population could therefore be used to detect QTL with the same analytical method used for an F_2_ population.

### QTL associated with pod shattering resistance in a RR-DH population

In the RR-DH population, four significant QTL *qSRI.A01a, qSRI.A06a, qSRI.A06b*, and *qSRI.A09* were detected for PSRI on chromosomes A01, A06, and A09 (Table 3). These QTL accounted for 5.66–16.91% of the phenotypic variation. The *qSRI.A09* (LOD = 4.31–7.69) accounted for the maximum phenotypic variation in pod shatter resistance (9.81–16.9%). Two QTL, *qSRI.A09* delimited with the SNP Bn-A09-p30171993 (A09) and *qSRI.A06b* delimited with the SNP marker Bn-A06-p115948 (A06) were repeatedly detected across both environments in 2013 and 2014. It is possible that QTL *qSRI.A06a* and *qSRI.A06b* may be the same, as both were detected in close proximity of Bn-A06-p15913910/Bn-A06-p115948 markers, mapped within 250 kb on the physical map of *B. napus* genome (Table 3, Supplementary Table 4). The pod shatter resistant parent, R1 contributed favorable alleles for pod shatter resistance based on RTI at all QTL detected (Table 3), consistent with the high pod shatter resistance index of R1 compared to R2 (Figure 1).

**Table 3:** **Comparison of QTL identified for pod shatter resistance from linkage mapping and association analysis of mapping populations**.

**Mapping population**	**Phenotyping year**	**QTL**	**Chr^a^**	**LOD**	***P*-Value for GWAS association**	**Lead SNP with highest *P*-value**	**Physical map position**	Genetic map position (cM)^*^	**Confident Interval**	***R*^2^ (%)**	**Candidate gene**
DH	2014	*qSRI.A01a*	A01	5.43		Bn-A01-p11702957	11221208	71.5	69.5–75.4	13.14	
IF_2_	2014	*qSRI.A01b*	A01	7.07		Bn-A01-p2365493	1858394	56.9	54.3–61.5	12.92	*SPATULA* (*SPT*)
GWAS		*qSRI.A01c*	A01		8E-06	Bn-A01-p10523833	883265	–	–	6.37	
IF_2_	2014	*qSRI.A03*	A03	3.84		Bn-scaff_22728_1-p75030	5375993	62	58.7–64.3	4.01	
DH	2013	*qSRI.A06a*	A06	2.93		Bn-A06-p15913910	17373387	60.4	56.2–63.4	5.66	*GIBBERELLIN 3-OXIDASE 1*
DH	2014	***qSRI.A06b***	**A06**	2.88		Bn-A06-p115948	79870	57.8	54–59.8	6.47	*GIBBERELLIN 3-OXIDASE 1*
IF_2_	2014	***qSRI.A06b***	**A06**	4.19		Bn-A06-p115948	79870	58.3	55.1–62.3	3.69	
GWAS		***qSRI.A06b***	**A06**		1.5E-05	Bn-A06-p115948	79870	–	–	6.61	
GWAS		*qSRI.A07*	A07		1E-06	Bn-A07-p7392457	858774	–	–	7.3	*YABBY1*
DH	2013	***qSRI.A09***	**A09**	7.69		Bn-A09-p30171993	3297223	68.1	65.8–75.9	16.91	*SHATTERPROOF 1/2, ARF18*
DH	2014	***qSRI.A09***	**A09**	4.31		Bn-A09-p30171993	3297223	68.1	67.4–76.5	9.81	
IF_2_	2014	***qSRI.A09***	**A09**	12.41		Bn-A09-p30171993	3297223	68.1	67.4–76.5	10.89	
GWAS		***qSRI.A09***	**A09**		4.4E-09	Bn-A09-p30171993	3297223	–	–	12.07	
GWAS		*qSRI.C02*	C02		6.5E-06	Bn-scaff_15712_6-p214229	40565480	–	–	6.32	
GWAS		*qSRI.C05*	C05		1.1E-06	Bn-scaff_17869_1-p1058624	19589640	–	–	7.23	

a Chromosome;

* *not applicable*.

*Consistent QTL identified across mapping populations/environments are in bold*.

### Verification of loci associated with pod shatter resistance in IF_2_ population

In order to verify the allelic effects of QTL revealed in a RR-DH population (Table 3), we performed an independent linkage analysis for association between SNP markers and genetic variation in pod shatter index evaluated in an IF_2_ population (Figure 1, Table 3). We identified four QTL, *qSRI.A01b, qSRI.A03, qSRI.A06b*, and *qSRI.A09* for PSRI on chromosomes A01, A03, A06, and A09, respectively (Table 3). Two consistent and stable QTL *qSRI.A06b and qSRI.A09*, as identified in RR-DH population, were also detected in an IF_2_ population. The same set of markers, Bn-A06-p115948 (A06) and Bn-A09-p30171993 (A09) revealed significant phenotypic variation for pod shatter resistance (Table 3). Significant QTL, *qSRI.A01b* (A01) and *qSRI.A03* (A03) were defined by the SNP markers Bn-A01-p2365493 and Bn-scaff-22728-1-p75030, respectively (Table 3). These QTL accounted for up to 13.14% of phenotypic variation in PSRI.

### GWAS analysis for pod shatter resistance in a diversity panel

In order to identify loci associated with pod shatter resistance in a diverse panel of accessions, exploiting the historic recombination events, we conducted a GWAS using the Q + K model accounting both for population structure as well as kinship relatedness (Bradbury et al., 2007). Based on a probability-of-membership (a measure of population structure) with threshold of 70%, a diversity panel of 143 lines could be assigned to three groups (group I: 17 lines, group II: 99 lines, and group III: 27 lines representing a mixed group; Supplementary Table 1). In addition, cluster analysis was conducted; the Neighbor-Joining phylogenetic tree based on Nei's genetic distances displayed two clear clades (Supplementary Figure 1), reconfirming the presence of two groups (group I and II, Supplementary Table 1) estimated by STRUCTURE. Estimates of an average nucleotide diversity (also known as polymorphism information content or PIC) of 0.366 showed that the overall genetic variation in the germplasms studied here represents ~62.9% of the rapeseed diversity (PIC > 0.35; Supplementary Table 2). In order to test the robustness of population structure revealed by cluster analysis, we also used the Δk method (Evanno et al., 2005). The 143 accessions could be divided into two sub-populations (Supplementary Figure 2). The average relative kinship between any two lines was 0.0332, or ~57% of the pairwise kinship estimates were close to 0, and 21% of the kinship estimates ranged from 0 to 0.05 (Supplementary Figure 3). The genome-wide LD decay of each chromosome for rapeseed germplasms is shown in Supplementary Figure 4.

GWAS detected a total of 38 SNPs that showed significant association (up to *P* < 2.90E^−5^) with pod shatter resistance across three environments (Table 3, Supplementary Table 3). After Bonferroni correction, we identified 6 genomic regions (QTL) on chromosomes A01, A06, A07, A09, C02, and C05 accounting for up to 45.9% cumulative phenotypic variance for pod shatter resistance in a GWAS panel (Table 3). Multiple environment analyses revealed that at least two QTL, *qSRI.A06b* delimited with the SNP marker Bn-A06-p115948 (A06) and *qSRI.A09* delimited with the SNP Bn-A09-p30171993 (A09) could be repeatedly detected across populations (DH, IF_2,_ and GWA panel) as shown in Table 3. Significant QTL associated with SNPs Bn-A07-p7392457 (A07), Bn-scaff_15712_6-p214229 (C02), and Bn-scaff_17869_1-p1058624 (C05) were not detected in both RR-DH/IF_2_ genetic mapping populations.

### Physical mapping of significant QTL for pod shatter resistance in comparison to previously detected QTL and candidate genes

**Figure 4:**
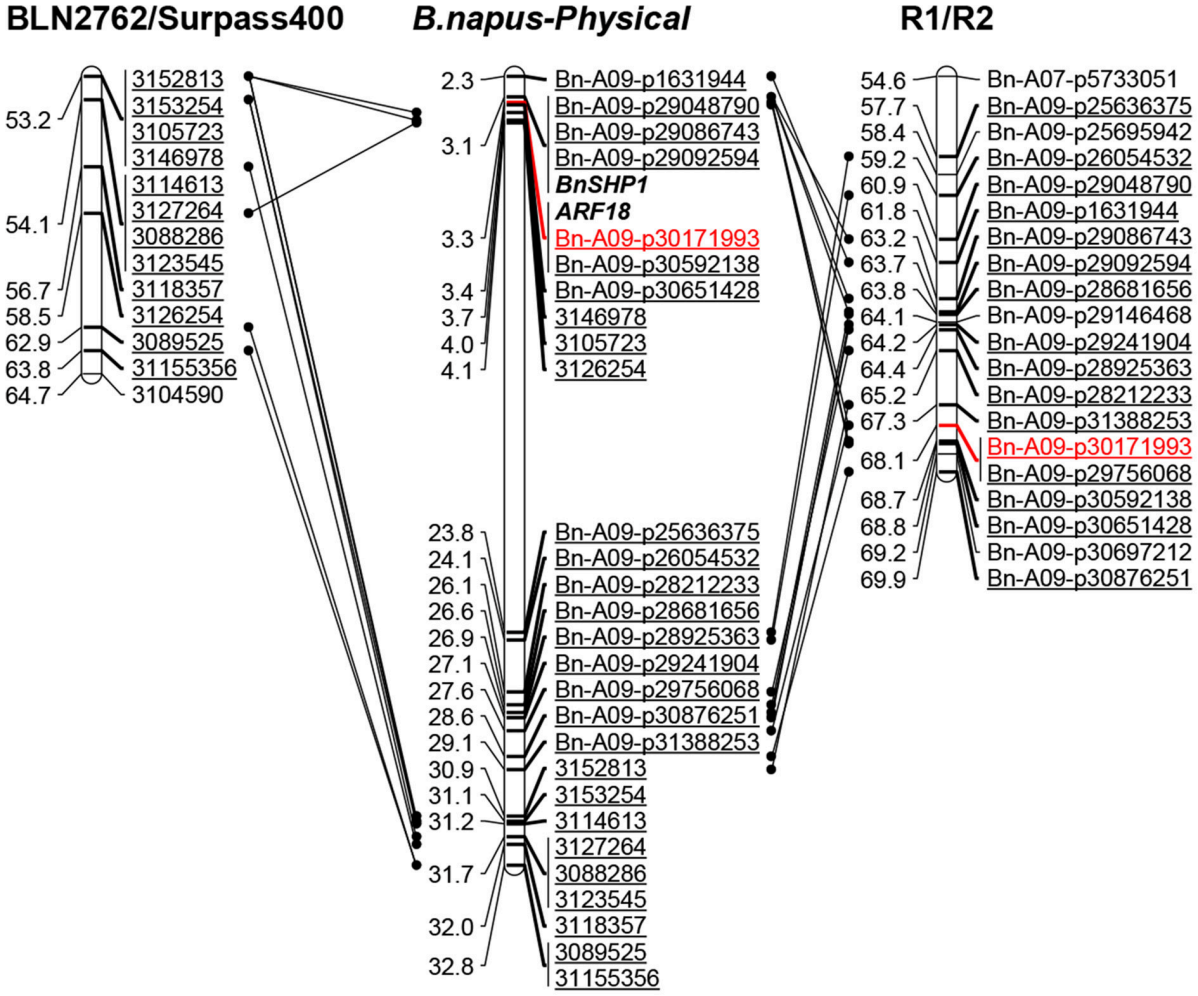
**Comparative analysis of ***qSRI.A09*** on chromosome A09 for pod shatter resistance in the RR-DH and previously mapped populations. Left:** the DArTseq markers in the order of the genetic map (cM) for *B. napus* based on a previous study (Raman et al., 2014). **Middle**: the markers in the order of the physical map (Kb) for *B. napus* (Darmor-bzh). Physical map distances are given in fraction (1/1,000,000th) of the actual coordinates of the *B. napus* genome. The markers in red are the most associated marker for pod shattering resistance. **Right**: the markers in the order of the genetic map (cM) for R1/R2 (RR-DH) population used in the current study. The marker in red showed highly significant association with pod shatter resistance at *qSRI.A09*.

In order to gain insights of genetic architecture of pod shatter resistance loci, we compared the physical positions of markers associated with QTL identified in this current and previously studies (Hu et al., 2012; Raman et al., 2014). The sequences of markers significantly associated with pod shatter resistance were subjected to BLAST against the physical reference genome of *B. napus*. The markers linked with pod shatter resistance loci on chromosome A09: NS380 and NS381 (Hu et al., 2012), DArTseq markers 3146978 and 3105723 (Raman et al., 2014) and Bn-A09-p30171993 (this study) were located within ~400 kb region of *B. napus* genome (Figure 4). This genomic region delimited from 30.84 to 31.98 Mb of *B. napus* genome also contains QTL having major allelic effects for pod length and seed weight in rapeseed (Li et al., 2014; Fu et al., 2015). A recent research showed that the *AUXIN RESPONSE FACTOR 18* (*ARF18*) gene affecting seed weight and pod length is located within this region (Liu et al., 2015). These studies suggested that *qSRI.A09* is a hotspot region for seed yield and pod traits such as pod shatter resistance and pod length in rapeseed. The major QTL genomic regions on A09 (Table 3) were consistent as reported previously, suggesting that indeed QTL identified herein are relevant to international germplasm and rapeseed breeding programs.

In order to identify putative candidate genes involved in pod shatter resistance in the mapping populations (GWAS, DH, and IF_2_) investigated herein, we compared the physical map positions of SNP markers that showed significant associations in GWAS and mapping populations and known candidate genes involved in positively and negatively regulation of pod shatter such as *FILAMENTOUS FLOWER, YABBY3, ASYMETERICAL LEAVES1/2, BREVIPEDICELLUS, SHATTERPROOF1/2, INDEHISCENT, ALCATRAZ, FRUITFUL, APETELA2, NAC SECONDARY WALL THICKENING PROMOTING FACTOR1, SECONDARY WALL-ASSOCIATED NAC DOMAIN PROTEIN1, DEHISCENCE ZONE POLYGALACTURONASE1, SPATULA*, and *PIN3* (reviewed in Dong and Wang, 2015) (Supplementary Table 4) on the sequenced *B. napus* genome. Among these significant SNPs underlying genetic variation for pod shatter resistance (Table 3), Bn-A01-p2365493 at the *qSRI.A01b* (A01) was mapped to a candidate gene *SPATULA;* Bn-A06-p15913910 and Bn-A06-p115948 corresponding with *qSRI.A06a* (A06) and the *qSRI.A06b* (A06) were all mapped to candidate genes *GIBBERELLIN 3-OXIDASE 1;* Bn-A07-p7392457 at the *qSRI.A07* (A07) was mapped to a candidate gene *YABBY1*. Except that, Bn-A09-p30171993 at the *qSRI.A09* (A09) was mapped two homologous regions on A09 and C08 which is within 11 kb from the *ARF18* gene controlling seed weight and pod length in *B. napus* (Liu et al., 2015). Both copies of *ARF18* in *B. napus*; *BnaA.ARF18.a* and *BnaA.ARF18.c* were also located on the physical positions of chromosomes A09 and C08, respectively (Supplementary Table 4, Figure 4). PCR marker, Shp-100925 associated with *BnSHP-1* locus on chromosome A09 was also mapped in the vicinity of *qSRI.A09* and *ARF18* (Figure 4).

### Allelic diversity at significant QTL associated with pod shatter resistance

Based on the PSRI ranking of 143 accessions used for GWAS, 18 elite cultivars having PSRI ≥ 0.28 were selected and their allele diversity was investigated at QTL *qSRI.A01, qSRI.A06b, qSRI.A07, qSRI.A09, qSRI.C02*, and *qSRI.C05* that showed significant associations with lead SNP markers (Tables 3, 4). These 18 accessions were originated from 5 provinces of China (Supplementary Table 1), representing the main rapeseed production area of the Yangtze River eco-region. About one-half of the resistant accessions, including the top five with PSRI ≥ 0.44 (Table 4), all originated from Hubei province in the middle Yangtze River eco-region, shared the “CC” SNP allele at Bn-A09-p30171993 locus. Generally, the resistant accessions possess multiple favorable alleles suggesting the potential for recombining them in a breeding design to improve resistance to pod shatter in rapeseed breeding programs. For example, the most resistant genotype, Zhongshuang2 might be further improved through complementary recombination with the favorable alleles (CC) of Bn-A09-p30171993 from other resistant accessions identified in this study (Table 4). In addition, combining favorable alleles among other accessions would also improve pod shatter resistance within a breeding program.

**Table 4:** **SNP alleles at the significant QTL identified for pod shatter resistance in DH, IF_**2,**_ and GWAS panel among 18 pod shatter resistant accessions**.

**Name**	**SRI mean**	**A01**		**A06**		**A07**		**A09**		**C02**		**C05**	
		**Bn-A01-p10523833 A/G**		**Bn-A06-p115948 A/G**		**Bn-A07-p7392457 T/G**		**Bn-A09-p30171993 A/C**		**Bn-scaff_15712_6-p214229 A/G**		**Bn-scaff_17869_1-p1058624 T/G**	
Zhongshuang2	0.49			AA		TT		AA		AA			GG
OG3151	0.47	AA			GG		GG		CC	AA		TT	
Zhen2609	0.46			AA					CC	AA			GG
R1	0.45			AA		TT			CC	AA			GG
OG3237	0.44	AA			GG		GG		CC	AA		TT	
9905	0.43		GG	AA					CC		GG		GG
OG3190	0.41				GG					AA			GG
HX0352	0.39						GG				GG	TT	
L229	0.38			AA			GG				GG		GG
1055B	0.35			AA			GG	AA		AA			GG
9490	0.35		GG	AA			GG		CC	AA			GG
9230	0.34	AA		AA			GG		CC	AA			GG
9226	0.31		GG	AA			GG		CC	AA			GG
Zhongshuang11	0.31			AA		TT			CC	AA			GG
L233	0.29			AA			GG			AA		TT	
Huyou19	0.29				GG				CC	AA		TT	
Fan189	0.28				GG			AC		AA			GG
OG3186	0.28				GG					AA			GG
*R*2	0.04	AA			GG		GG	AA			GG	TT	

## Discussion

### Genetic variation for pod shatter resistance in rapeseed

In this study, we determined the extent of genetic variation for pod shatter resistance in bi-parental DH and IF_2_ populations, and GWAS diversity panel comprising 143 accessions representing released Chinese cultivars/advanced breeding lines. We identified seven accessions with PSRI ≥ 0.4 across years which exhibited improved levels of PSRI such as Zhongshuang2, OG3151, and Zhen2609, compared to standard check cultivars and would provide valuable resources for genetic improvement of pod shatter resistance in rapeseed improvement programs. However, we could not benchmark the level of resistance to pod shatter among accessions utilized in this study and previous ones (Wen et al., 2008; Pu et al., 2013, Raman et al., 2014), due to different assessment methods, germplasm, and growing conditions. Previous studies showed that there is a limited natural variation for pod shatter resistance in rapeseed (Wen et al., 2008; Raman et al., 2014), which has contributed to the lack of significant genetic improvement for this trait in breeding programs. It is possible that improved pod shatter resistance characterized herein may have been derived from pod shatter resistant sources of *B. rapa*, as they have been extensively used for introgression of novel alleles for traits of interest as well as to expand genetic base of rapeseed germplasm especially in China (Qian et al., 2005; Zou et al., 2010). Sources of pod shatter resistance are well documented in *B. rapa* gene pool and have been exploited in breeding programs (Kadkol et al., 1985, 1986; Mongkolporn et al., 2003; Hossain et al., 2011; Raman et al., 2014).

A laboratory based method (RIT) proved to be robust in determining the extent of pod-shatter resistance across several experiments. Further research efforts are needed to validate RIT for pod shatter resistance with pendulum test and field based methods such as delayed harvest across rapeseed growing regions.

### Genetic basis of phenotypic variation in pod shatter resistance

We utilized both classical QTL and GWAS approaches to detect genomic regions associated with pod shatter resistance (Table 3). Both these approaches have their own advantages and disadvantages in QTL detection. For example, classical linkage analysis has strong statistical power and proven to be effective in detecting QTL, but only capture the recombination events in two parents used in constructing bi-parental DH/intercross populations. GWA simultaneously detects multiple alleles at the same locus, due to the accumulation of historical recombination events during systematic selection in breeding and resolves QTL based on LD particularly in species such as rapeseed where LD decays rapidly (Flint-Garcia et al., 2003; Buckler et al., 2009; Gajardo et al., 2015). The combined application of both approaches; QTL and GWAS not only improve the efficiency of QTL detection, but also facilitate the identification of reliable and stable QTL and novel alleles across a wide range of germplasm (Krill et al., 2010; Raman et al., 2014, 2016).

In this study, we identified six QTL associated with pod shatter resistance which accounted for up to 50% the phenotypic variation in PSRI in DH and IF_2_ mapping populations. Previously, several QTL associated with pod shatter resistance were identified in a DH mapping populations derived from ZY72360/R1, H155/Qva, and BLN2762/Surpass400, and in diverse panel of accessions of *B. napus*, originated from Australia, China, and Europe (Hu et al., 2012; Wen et al., 2013; Raman et al., 2014). For example, Wen et al. (2013) identified 13 QTL for pod shatter resistance on the chromosomes A01, A04, A07, A08, C05, and C08; however only three of them were consistent at both locations. Recently, Raman et al. (2014) identified 12 QTL associated with pod shatter resistance in a DH population from BLN2762/Surpass400 on chromosomes A03, A07, A09, C03, C04, C06, and C08 using DArTseq markers. *In silico* mapping analysis of Illumina SNP markers showed that some of the QTL identified in this study are similar as reported previously (Raman et al., 2014) such as on A01, A03, and A09. Two QTL *qSRI.A06* (A06) and *qSRI.A09* (A09) were detected repeatedly across DH and GWAS populations and phenotypic environments, implicating their involvement in pod shatter resistance in rapeseed cultivars of Chinese origin. This suggests that there were at least two genes involved in resistance to pod shattering in DH and IF_2_ populations derived from R1. In a previous study (Hu et al., 2012), one major quantitative trait locus *psr1* on chromosome A09 accounting 47% of phenotypic variation in pod shatter resistance was identified in an F_2_ population derived from ZY72360/R1. Comparative analysis of the A09 locus in the linkage maps of BLN2762/Surpass400 (Raman et al., 2014) and R1/R2 (this study) with *B. napus* physical map, showed an inversion event of the 400 kb QTL interval *qSRI.A09*/*Qrps.wwai-A09*. This result is partly consistent with the previous comparative genomic studies showing rearrangements in the A subgenome of *B. napus* (Xu et al., 2010; Li et al., 2014).

The present study showed that the PCR marker, Shp-100925 associated with *BnSHP-1* locus was mapped in the vicinity of *qSRI.A09* and *ARF18* (Figure 4). The role of auxin in pod dehiscence and other developmental processes has been documented in Arabidopsis (Okushima et al., 2005; Sorefan et al., 2009), *B. juncea*, and *B. napus* (Jaradat et al., 2014). For example, Sorefan et al. (2009) reported that a local auxin minimum is required for the formation of valve margin separation layer for seed dehiscence which is controlled by *IND* gene. *ARF18* gene also regulates cell growth in the pod wall via auxin-response pathway in *B. napus* and simultaneously affects seed weight and pod length in an F_2_ population derived from the ZY72360/R1 (Liu et al., 2015). In a recent study, auxin biosynthesis, transport, and signaling was shown to be repressed in *B. juncea* (less prone to shattering) compared to *B. napus* (more prone to pod shattering) genotypes (Jaradat et al., 2014). These studies suggest that that the auxin minimum may be responsible for pod shatter trait in the mapping populations investigated here. Further studies are required to establish the role of auxins in genetic variation for pod shattering resistance in diverse *B. napus* accessions.

In addition to *qSRI.A09*/*Qrps.wwai-A09*/*psr1* locus on A09 (Hu et al., 2012; Raman et al., 2014, this study), other QTL *qSRI.A01* (A01), *qSRI.A03* (A03), *qSRI.A07* (A07), *qSRI.C02* (C02), and *qSRI.C05* (C05) also account genetic variation for pod shatter resistance derived from R1, a pod shatter resistant Chinese cultivar. Arabidopsis genes underlying the significant QTL such as *SPATULA* and *GIBBERELLIN 3-OXIDASE 1* (Table 3) are likely candidate genes for pod shatter resistance in mapping populations. A basic-helix-loop-helix transcription factor, *SPATULA* is implicated in dehiscence zone in Arabidopsis and regulated by *ARF* (Heisler et al., 2001), suggesting its role in auxin-mediated dehiscence zone formation implicated in pod shatter. GA3ox1 encodes a Gibberellin 3-oxidase, which is a direct and necessary target of *IND* gene (Arnaud et al., 2010). Identification of closely linked markers and the genomic location of QTL on chromosomes A01, A06, A07 and A09 with respect to a reference genome of *B. napus* and the described genes involved in pod shatter resistance of Arabidopsis could also pave the way for map-based cloning of those QTL and unravel the molecular architecture of pod shatter resistance genes in natural germplasm of *B. napus*.

## Conclusion

Both GWAS and linkage analyses enabled to untangle multiple quantitative trait loci associated with pod shatter resistance in Chinese germplasm of rapeseed. Identification of the improved sources for pod shatter resistance, and understanding the genetic basis underlying genetic variation in pod shattering resistance in rapeseed germplasm will provide insights into the complex architecture and evolution of this trait which has been subjected to artificial selection since its domestication. SNP markers flanking QTL regions would provide an efficient method for selection of alleles associated with pod shatter resistance in rapeseed breeding programs.

## Author contributions

JL and QH conceived and designed the study. JW and HW conducted the DH and IF_2_ population experiments; JL and WW carried out the association population experiments; JW and HW analyzed the DH and IF_2_ data; JL and WW analyzed the association data; DM and JW produced the DH and IF_2_ populations; RZ, HC, and JY did the phenotype assessment; JL, JW, and HR interpreted the data and prepared the manuscript; HR performed comparative and *in silico* analysis; QH supervised the whole study; all authors reviewed and edited the manuscript.

### Conflict of interest statement

The authors declare that the research was conducted in the absence of any commercial or financial relationships that could be construed as a potential conflict of interest.

## Footnotes

^1^Oilseeds: World Markets and Trade|USDA FAS. Fas.usda.gov. Retrieved 2015-08-25.
