# Supplementary material for: Multigenic Control of Pod Shattering Resistance in Chinese Rapeseed Germplasm Revealed by Genome-Wide Association and Linkage Analyses
Source: Front Plant Sci. 2016 Jul 21;7:1058. doi: 10.3389/fpls.2016.01058 (PMC4954820; doi:10.3389/fpls.2016.01058)
Supplement: Supplementary file 2 [file DataSheet1.DOCX]

Supplementary Material

**Multigenic control of pod shatter resistance in Chinese rapeseed germplasm revealed by genome-wide association and linkage analyses**

# Supplementary Figures and Tables

## Supplementary Figures


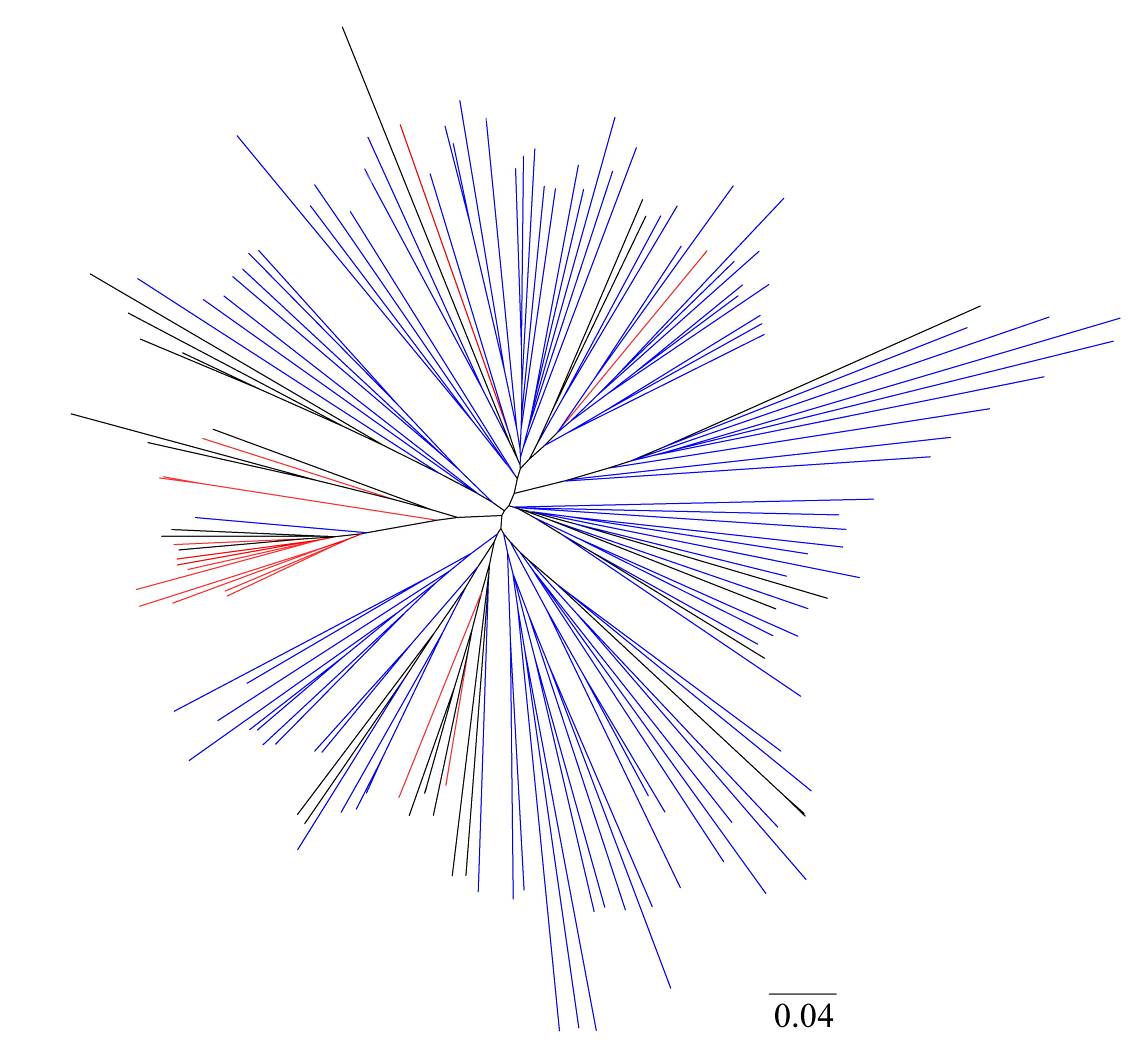


**Supplementary Figure 1.** A neighbor-joining phylogenetic tree based on Nei’s genetic distance. Red states for Group 1, blue for Group 2, and black for the mixed group.


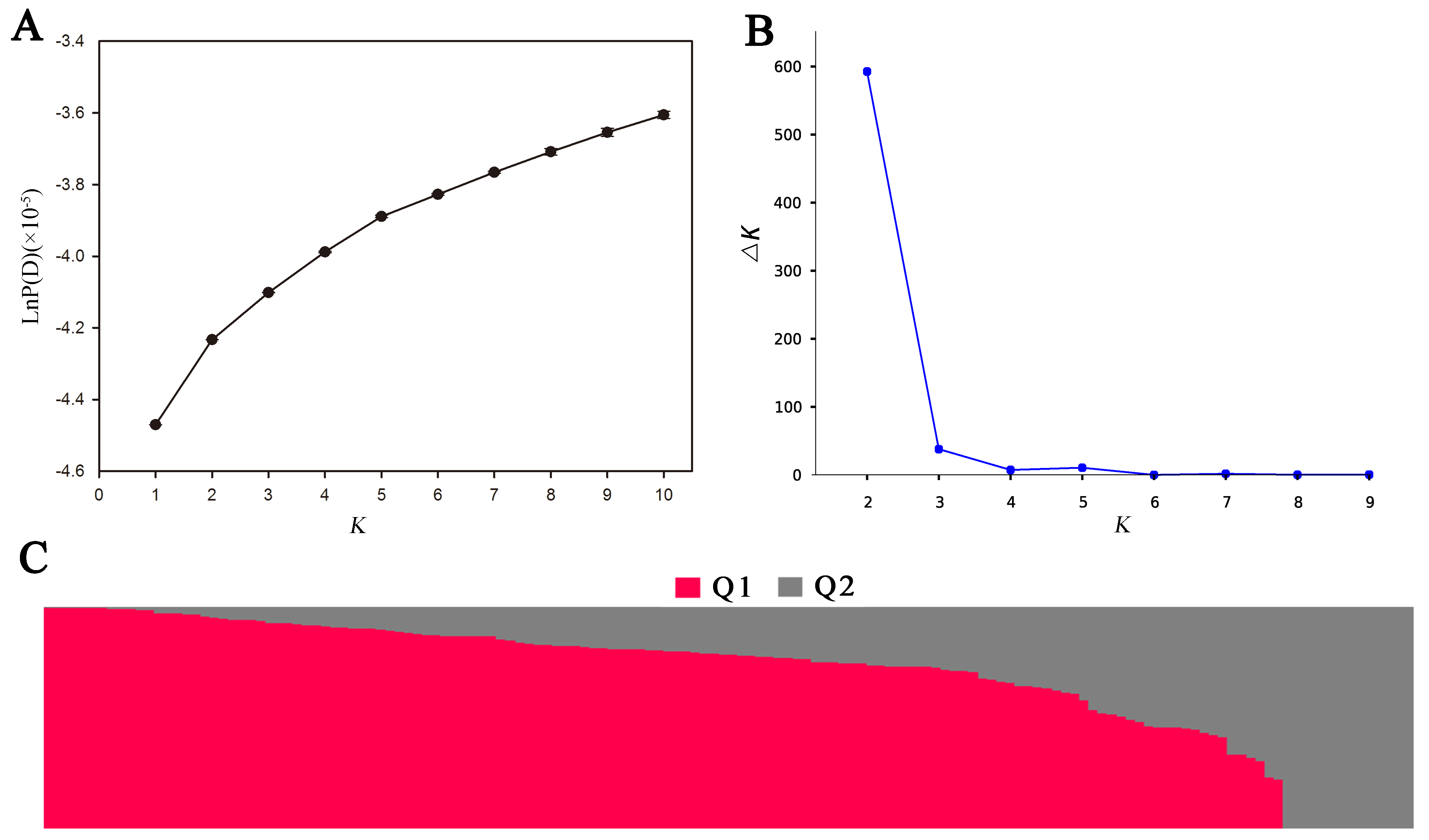


**Supplementary Figure 2.** Population structure analysis of 143 rapeseed germplasms by STRUCTURE software. (a) The estimated LnP(D) of possible clusters (k) from 1 to 10; (b) ∆k based on the change of LnP(D) between consecutive k; and (c) Q1 and Q2 are the composition values belonging to the two sub-populations (*K* = 2) for a given germplasm which is represented by a vertical bar.


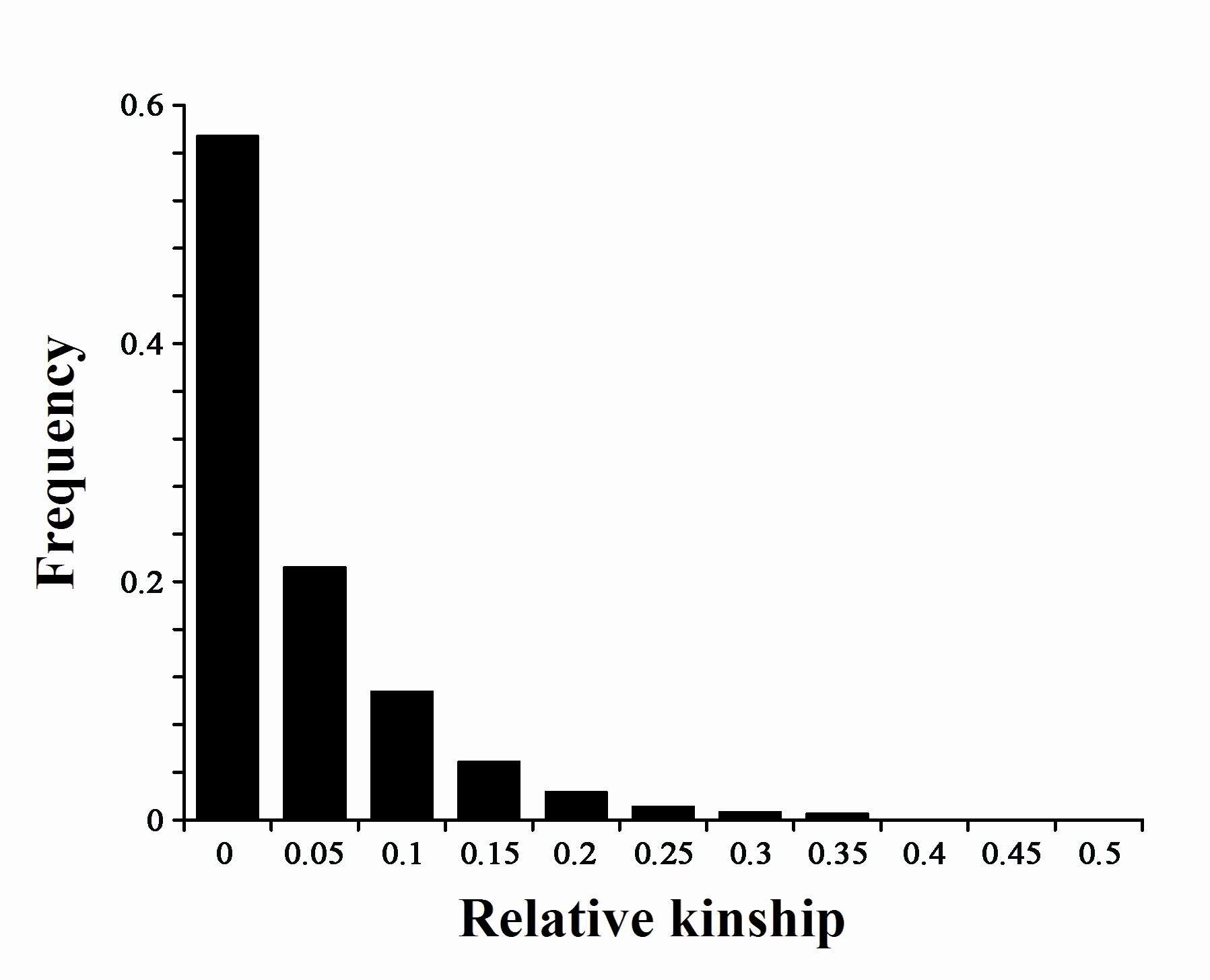


**Supplementary Figure 3.** The distribution for pairwise relative kinship. Only the kinship values ranging from 0 to 0.57 are shown.


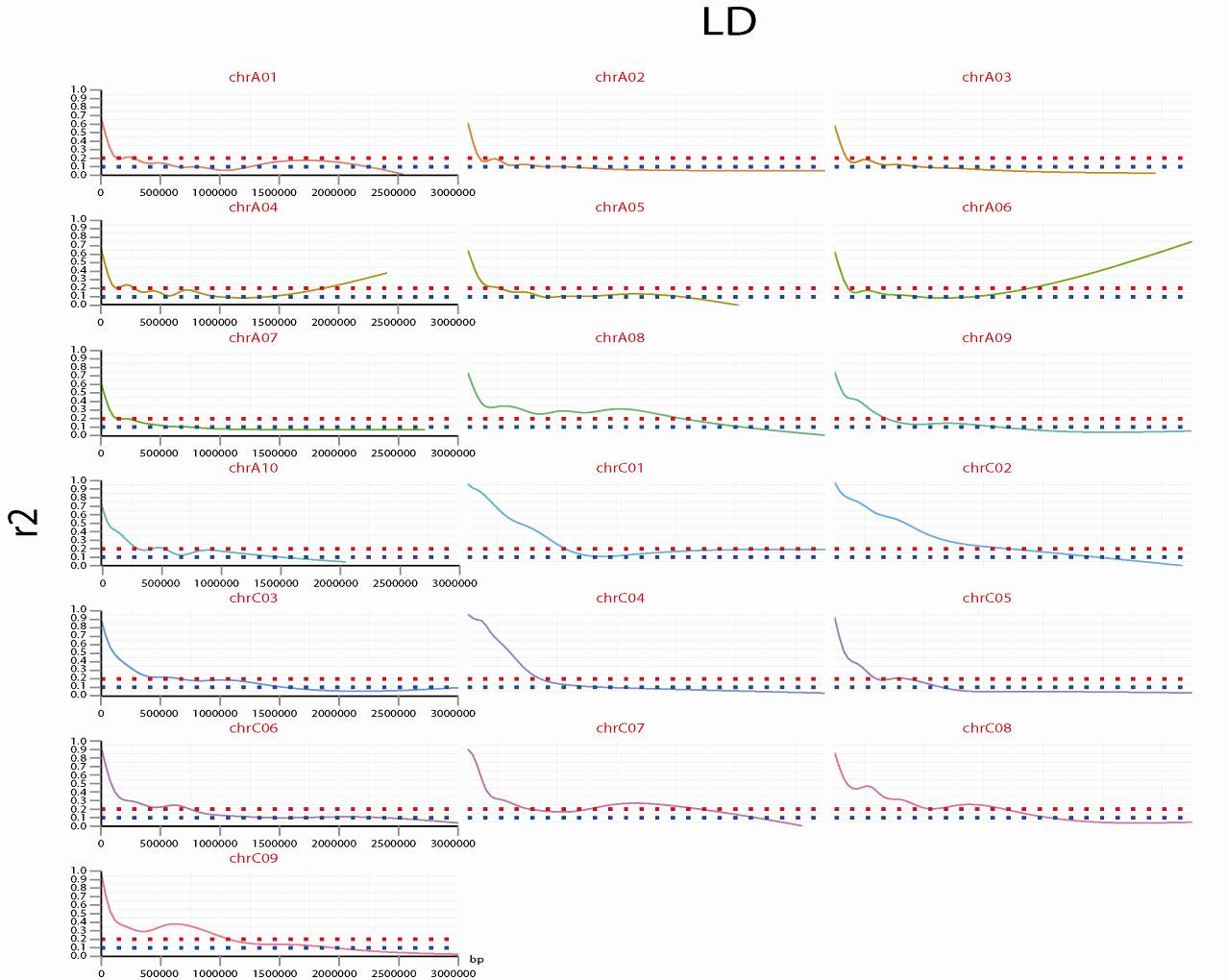


**Supplementary Figure 4.** Genome-wide LD decay of A01-A10 and C01-C09 genomes for all the 143 accessions.

## Supplementary Tables

**Supplementary Table 1.** A list of *B. napus* germplasm lines used for association study.

| **Accession number** | **Inbred lines** | **Country/Region** | **Type** | **Cluster^*^** |
| --- | --- | --- | --- | --- |
| 1 | 0028 | Hubei (China) | Semi-winter OSR | Group2 |
| 2 | R6 | Hubei (China) | Semi-winter OSR | Group2 |
| 3 | 1019B | Hubei (China) | Semi-winter OSR | Group2 |
| 4 | R9 | Hubei (China) | Semi-winter OSR | Group2 |
| 5 | 1055B | Hubei (China) | Semi-winter OSR | Group2 |
| 6 | Chuan2121 | Hubei (China) | Semi-winter OSR | Group2 |
| 7 | 6098B | Hubei (China) | Semi-winter OSR | Group2 |
| 8 | HPR1 | Hubei (China) | Semi-winter OSR | Group1 |
| 9 | Zayou66 | Hubei (China) | Semi-winter OSR | Group1 |
| 10 | K219 | Hubei (China) | Semi-winter OSR | Group1 |
| 11 | 8089 | Hubei (China) | Semi-winter OSR | Group2 |
| 12 | Shan2C | Shanxi (China) | Winter | Mix |
| 13 | 8908B | Hubei (China) | Semi-winter OSR | Group2 |
| 14 | Zhongshuang2 | Hubei (China) | Semi-winter OSR | Group2 |
| 15 | Shan2B | Shanxi (China) | Winter | Group2 |
| 16 | Zhongshuang6 | Hubei (China) | Semi-winter OSR | Group2 |
| 17 | R1 | Hubei (China) | Semi-winter OSR | Group2 |
| 18 | R11 | Hubei (China) | Semi-winter OSR | Group2 |
| 19 | R2 | Hubei (China) | Semi-winter OSR | Group2 |
| 20 | Zhongshuang11 | Hubei (China) | Semi-winter OSR | Mix |
| 21 | Nilla | European | Winter | Group1 |
| 22 | OG3186 | Hubei (China) | Semi-winter OSR | Group2 |
| 23 | Cibrabra | European | Winter | Group2 |
| 24 | OG3237 | Hubei (China) | Semi-winter OSR | Mix |
| 25 | Apomix | European | Winter | Group2 |
| 26 | OG3151 | Hubei (China) | Semi-winter OSR | Group2 |
| 27 | OG2097 | Hubei (China) | Semi-winter OSR | Mix |
| 28 | OG3156 | Hubei (China) | Semi-winter OSR | Group2 |
| 29 | OG2086 | Hubei (China) | Semi-winter OSR | Mix |
| 30 | OG3174 | Hubei (China) | Semi-winter OSR | Group2 |
| 31 | OG2118 | Hubei (China) | Semi-winter OSR | Mix |
| 32 | OG3183 | Hubei (China) | Semi-winter OSR | Mix |
| 33 | OG3229 | Hubei (China) | Semi-winter OSR | Mix |
| 34 | Shilifeng | Jiangsu (China) | Semi-winter OSR | Group2 |
| 35 | OG3187 | Hubei (China) | Semi-winter OSR | Group2 |
| 36 | Oscar | Australia | Semi-winter OSR | Mix |
| 37 | OG3190 | Hubei (China) | Semi-winter OSR | Group1 |
| 38 | Surpass400 | Australia | Semi-winter OSR | Mix |
| 39 | OG3191 | Hubei (China) | Semi-winter OSR | Group2 |
| 40 | Hektor | European | Winter | Mix |
| 41 | Zheyou50 | Zhejiang (China) | Semi-winter OSR | Group2 |
| 42 | HM42 | Zhejiang (China) | Semi-winter OSR | Group2 |
| 43 | Huashuang5 | Hubei (China) | Semi-winter OSR | Group2 |
| 44 | Huyou17 | Shanghai (China) | Semi-winter OSR | Group2 |
| 45 | L233 | Chongqing (China) | Semi-winter OSR | Group2 |
| 46 | Rivette | Australia | Semi-winter OSR | Group2 |
| 47 | RR001 | Australia | Semi-winter OSR | Group2 |
| 48 | 0331-1 | Hubei (China) | Semi-winter OSR | Group2 |
| 49 | Zheyou21 | Zhejiang (China) | Semi-winter OSR | Group2 |
| 50 | Zhongshuang4 | Hubei (China) | Semi-winter OSR | Group2 |
| 51 | Zheshuang6 | Zhejiang (China) | Semi-winter OSR | Group2 |
| 52 | Zhongshuang10 | Hubei (China) | Semi-winter OSR | Group2 |
| 53 | Zheyou28 | Zhejiang (China) | Semi-winter OSR | Group2 |
| 54 | Zheshuang72 | Zhejiang (China) | Semi-winter OSR | Group2 |
| 55 | Westar | Canada | Spring | Group2 |
| 56 | Zheshuang8 | Zhejiang (China) | Semi-winter OSR | Group2 |
| 57 | BLN3344 | Australia | Spring | Group2 |
| 58 | BLN3346 | Australia | Spring | Group2 |
| 59 | BLN3350 | Australia | Spring | Group2 |
| 60 | BLN3352 | Australia | Spring | Group2 |
| 61 | RT006 | Australia | Spring | Group2 |
| 62 | RT108 | Australia | Spring | Group2 |
| 63 | RT123 | Australia | Spring | Group2 |
| 64 | FC03 | Australia | Spring | Mix |
| 65 | Huyou21 | Shanghai (China) | Semi-winter OSR | Group2 |
| 66 | （R1-1*V）F7 | Hubei (China) | Semi-winter OSR | Group2 |
| 67 | YangJ6711 | Jiangsu (China) | Semi-winter OSR | Group2 |
| 68 | Zhen2736 | Jiangsu (China) | Semi-winter OSR | Group2 |
| 69 | HX0352 | Hubei (China) | Semi-winter OSR | Group2 |
| 70 | BLN3342 | Australia | Spring | Mix |
| 71 | Zhen2609 | Jiangsu (China) | Semi-winter OSR | Group2 |
| 72 | Zhen3736 | Hubei (China) | Semi-winter OSR | Group2 |
| 73 | Ningyou16 | Jiangsu (China) | Semi-winter OSR | Group2 |
| 74 | Zhongyou821 | Hubei (China) | Semi-winter OSR | Group2 |
| 75 | Ningyou18 | Jiangsu (China) | Semi-winter OSR | Group2 |
| 76 | Fan168 | Hubei (China) | Semi-winter OSR | Group2 |
| 77 | Zhenyou5 | Jiangsu (China) | Semi-winter OSR | Group2 |
| 78 | Fan189 | Hubei (China) | Semi-winter OSR | Group2 |
| 79 | Yangyou4 | Jiangsu (China) | Semi-winter OSR | Group2 |
| 80 | J110 | Hubei (China) | Semi-winter OSR | Group2 |
| 81 | Yangyou6 | Jiangsu (China) | Semi-winter OSR | Group2 |
| 82 | J474 | Hubei (China) | Semi-winter OSR | Group2 |
| 83 | Huyou15 | Shanghai (China) | Semi-winter OSR | Group2 |
| 84 | Zhongshuang7 | Hubei (China) | Semi-winter OSR | Group2 |
| 85 | Huyou16 | Shanghai (China) | Semi-winter OSR | Group2 |
| 86 | Zhongshuang9 | Hubei (China) | Semi-winter OSR | Group2 |
| 87 | Huyou19 | Shanghai (China) | Semi-winter OSR | MiX |
| 88 | Zheyou18 | Zhejiang (China) | Semi-winter OSR | Group2 |
| 89 | P10 | Chongqing (China) | Spring | Group1 |
| 90 | 901 | Hubei (China) | Semi-winter OSR | Mix |
| 91 | P96 | Chongqing (China) | Spring | Mix |
| 92 | Huashuang3 | Hubei (China) | Semi-winter OSR | Group2 |
| 93 | P130 | Chongqing (China) | Spring | Group2 |
| 94 | Huashuang4 | Hubei (China) | Semi-winter OSR | Group2 |
| 95 | F403 | Chongqing (China) | Semi-winter OSR | Group2 |
| 96 | F406 | Chongqing (China) | Semi-winter OSR | Group1 |
| 97 | RR013 | Australia | Semi-winter OSR | Group2 |
| 98 | F421 | Chongqing (China) | Semi-winter OSR | Mix |
| 99 | Trigold | Australia | Semi-winter OSR | Mix |
| 100 | F426 | Chongqing (China) | Semi-winter OSR | Group2 |
| 101 | RR005 | Australia | Semi-winter OSR | Group1 |
| 102 | F428 | Chongqing (China) | Semi-winter OSR | Group2 |
| 103 | AV-SAPPHIRE | Australia | Semi-winter OSR | Group2 |
| 104 | F437 | Chongqing (China) | Semi-winter OSR | Group2 |
| 105 | AG-outback | Australia | Semi-winter OSR | Group2 |
| 106 | F459 | Chongqing (China) | Semi-winter OSR | Mix |
| 107 | Purler | Australia | Semi-winter OSR | Mix |
| 108 | L239 | Chongqing (China) | Semi-winter OSR | Mix |
| 109 | RR002 | Australia | Semi-winter OSR | Group2 |
| 110 | L238 | Chongqing (China) | Semi-winter OSR | Mix |
| 111 | GSL2 | India | Semi-winter OSR | Group2 |
| 112 | L230 | Chongqing (China) | Semi-winter OSR | Group2 |
| 113 | Monty | Australia | Semi-winter OSR | Group2 |
| 114 | L229 | Chongqing (China) | Semi-winter OSR | Group2 |
| 115 | TQ055-02W2 | Australia | Semi-winter OSR | Group2 |
| 116 | L228 | Chongqing (China) | Semi-winter OSR | Group2 |
| 117 | Skipton | Australia | Semi-winter OSR | Group2 |
| 118 | BST7-02M2 | Australia | Semi-winter OSR | Group2 |
| 119 | 0594-1 | Hubei (China) | Semi-winter OSR | Group2 |
| 120 | 0789-3 | Hubei (China) | Semi-winter OSR | Group1 |
| 121 | 9490-1 | Hubei (China) | Semi-winter OSR | Group2 |
| 122 | 0802-1 | Hubei (China) | Semi-winter OSR | Group2 |
| 123 | 9502-10 | Hubei (China) | Semi-winter OSR | Group2 |
| 124 | 0832-1 | Hubei (China) | Semi-winter OSR | Group2 |
| 125 | 9558-1 | Hubei (China) | Semi-winter OSR | Mix |
| 126 | 2731-3 | Hubei (China) | Semi-winter OSR | Group2 |
| 127 | 9559-30 | Hubei (China) | Semi-winter OSR | Group2 |
| 128 | 8596-3 | Hubei (China) | Semi-winter OSR | Group2 |
| 129 | 9905-4 | Hubei (China) | Semi-winter OSR | Group2 |
| 130 | 8599-3 | Hubei (China) | Semi-winter OSR | Group1 |
| 131 | 9906-1 | Hubei (China) | Semi-winter OSR | Mix |
| 132 | 9016-1 | Hubei (China) | Semi-winter OSR | Group1 |
| 133 | Wanyou12 | Anhui (China) | Semi-winter OSR | Group2 |
| 134 | 9022-5 | Hubei (China) | Semi-winter OSR | Mix |
| 135 | Wanyou20 | Anhui (China) | Semi-winter OSR | Mix |
| 136 | 9226-3 | Hubei (China) | Semi-winter OSR | Group1 |
| 137 | Zheshuang3 | Zhejiang (China) | Semi-winter OSR | Mix |
| 138 | 9230-5 | Hubei (China) | Semi-winter OSR | Group1 |
| 139 | Zheshuang758 | Zhejiang (China) | Semi-winter OSR | Group1 |
| 140 | 9386-1 | Hubei (China) | Semi-winter OSR | Group1 |
| 141 | NY 7 | Jiangsu (China) | Semi-winter OSR | Group1 |
| 142 | 9388-2 | Hubei (China) | Semi-winter OSR | Group2 |
| 143 | Matador | European | Semi-winter OSR | Group1 |

* Clusters were defined by STRUCTURE packate when k=2

| **Supplementary Table 2.** Summary of the PIC values in different linkage groups of *B. napus* | | | | | | | | | |  | |  |  |  | |  |  |  |
| --- | --- | --- | --- | --- | --- | --- | --- | --- | --- | --- | --- | --- | --- | --- | --- | --- | --- | --- |
| Linkage  group | Number  of SNPs | PIC^a^  Average |  |  |  |  |  | PIC value |  | |  | |  |  |  | |  | |
|  |  |  | 0.05-0.1 | 0.1-0.15 | 0.15-0.2 | 0.2-0.25 | 0.25-0.3 | 0.3-0.35 | 0.35-0.4 | | 0.4-0.45 | | 0.45-0.5 | 0.5-0.55 | 0.55-0.6 | | Marker  density(kb) | |
| A01 | 1868 | 0.389 | 5(0.3%) | 45(2.4%) | 113(6.1%) | 117(6.3%) | 121(6.5%) | 156(8.6%) | 251(13.4%) | | 473(25.3%) | | 323(17.3%) | 159(8.5%) | 105(5.6%) | | 14.6 | |
| A02 | 1691 | 0.372 | 3(0.2%) | 35(2.1%) | 95(5.6%) | 103(6.1%) | 186(11.0%) | 186(11.0%) | 360(21.3%) | | 381(22.5%) | | 167(9.9%) | 107(6.3%) | 68(4.0%) | | 15.4 | |
| A03 | 2267 | 0.372 | 5(0.2%) | 77(3.4%) | 86(3.8%) | 133(5.9%) | 212(9.4%) | 287(12.7%) | 453(20.0%) | | 561(24.8%) | | 247(10.9%) | 150(6.6%) | 56(2.5%) | | 13.9 | |
| A04 | 1541 | 0.370 | 2(0.1%) | 39(2.5%) | 60(3.9%) | 138(9.0%) | 131(8.5%) | 168(10.9%) | 317(20.6%) | | 402(26.1%) | | 163(10.6%) | 72(4.7%) | 48(3.1%) | | 13.2 | |
| A05 | 1694 | 0.369 | 6(0.4%) | 44(2.6%) | 91(5.4%) | 112(6.6%) | 154(9.1%) | 201(11.9%) | 366(21.6%) | | 353(20.8%) | | 218(12.9%) | 84(5.0%) | 65(3.8%) | | 14.1 | |
| A06 | 1532 | 0.376 | 0(0.0%) | 36(2.4%) | 64(4.2%) | 112(7.3%) | 98(6.4%) | 159(10.4%) | 344(22.5%) | | 415(27.1%) | | 203(13.3%) | 62(4.1%) | 39(2.6%) | | 16.9 | |
| A07 | 2093 | 0.375 | 8(0.4%) | 51(2.4%) | 91(4.4%) | 170(8.1%) | 166(7.9%) | 219(10.5%) | 382(18.3%) | | 543(25.9%) | | 244(11.7%) | 168(8.0%) | 51(2.4%) | | 12.3 | |
| A08 | 1055 | 0.325 | 3(0.3%) | 76(7.2%) | 92(8.7%) | 116(11.0%) | 181(17.2%) | 108(10.2%) | 147(13.9%) | | 204(19.3%) | | 76(7.2%) | 36(3.4%) | 16(1.5%) | | 17.6 | |
| A09 | 2114 | 0.397 | 4(0.2%) | 57(2.7%) | 74(3.5%) | 83(3.9%) | 159(7.5%) | 204(9.7%) | 334(15.8%) | | 452(21.4%) | | 493(23.3%) | 169(8.0%) | 85(4.0%) | | 19.0 | |
| A10 | 1212 | 0.385 | 5(0.4%) | 46(3.8%) | 47(3.9%) | 63(5.2%) | 85(7.0%) | 116(9.6%) | 173(14.3%) | | 332(27.4%) | | 257(21.2%) | 48(4.0%) | 40(3.3%) | | 12.1 | |
| C01 | 2497 | 0.348 | 21(0.8%) | 99(4.0%) | 77(3.1%) | 221(8.9%) | 156(6.3%) | 235(9.4%) | 1167(46.7%) | | 274(11.0%) | | 150(6.0%) | 50(2.0%) | 47(1.9%) | | 19.5 | |
| C02 | 1842 | 0.365 | 2(0.1%) | 20(1.1%) | 30(1.6%) | 106(5.8%) | 234(12.7%) | 407(22.1%) | 329(17.9%) | | 455(24.7%) | | 193(10.5%) | 45(2.4%) | 20(1.1%) | | 28.1 | |
| C03 | 2745 | 0.343 | 2(0.1%) | 168(6.1%) | 158(5.8%) | 180(6.6%) | 343(12.5%) | 387(14.1%) | 592(21.6%) | | 617(22.5%) | | 193(7.0%) | 59(2.2%) | 46(1.7%) | | 26.4 | |
| C04 | 2542 | 0.383 | 24(0.9%) | 85(3.3%) | 40(1.6%) | 134(5.3%) | 135(5.3%) | 197(7.8%) | 582(22.9%) | | 899(35.4%) | | 354(13.9%) | 58(2.3%) | 34(1.3%) | | 21.6 | |
| C05 | 932 | 0.355 | 1(0.1%) | 39(4.2%) | 43(4.6%) | 59(6.3%) | 91(9.8%) | 163(17.5%) | 237(25.4%) | | 154(16.5%) | | 108(11.6%) | 18(1.9%) | 19(2.0%) | | 47.6 | |
| C06 | 1892 | 0.363 | 1(0.1%) | 101(5.3%) | 78(4.1%) | 108(5.7%) | 96(5.1%) | 418(22.1%) | 254(13.4%) | | 369(19.5%) | | 417(22.0%) | 31(1.6%) | 19(1.0%) | | 24.7 | |
| C07 | 2383 | 0.360 | 1(0.0%) | 69(2.9%) | 47(1.9%) | 203(8.5%) | 574(24.1%) | 178(7.5%) | 274(11.5%) | | 384(16.1%) | | 500(21.0%) | 129(5.4%) | 24(1.0%) | | 22.6 | |
| C08 | 1407 | 0.355 | 0(0.0%) | 34(2.4%) | 26(1.9%) | 250(17.8%) | 176(12.5%) | 128(9.1%) | 238(16.9%) | | 299(21.3%) | | 191(13.6%) | 43(3.1%) | 22(1.6%) | | 26.6 | |
| C09 | 1189 | 0.346 | 13(1.1%) | 90(7.6%) | 35(2.9%) | 93(7.8%) | 80(6.7%) | 319(26.8%) | 109(9.2%) | | 181(15.2%) | | 219(18.4%) | 28(2.4%) | 22(1.9%) | | 52.6 | |
| Total | 34496 | **0.366** | 106(0.3%) | 1211(3.5%) | 1347(3.9%) | 2501(7.3%) | 3378(9.8%) | 4236(12.3%) | 6909(20.0%) | | 7748(22.5%) | | 4716(13.7%) | 1516(4.4%) | 826(2.4%) | | 22.0 | |

^a^PIC is an abbreviation of polymorphism information content

**Supplementary Table 3.** Summary of 38 SNPs that showed significant association (*P* <2.90E^-5^) with pod shatter resistance across three environments by GWAS.

| QTL | Marker | Chromosome | Site | p |
| --- | --- | --- | --- | --- |
| *qSRI.A01c* | Bn-A01-p10523833 | A01 | 10042324 | 8.04E-06 |
| *qSRI.A06b* | Bn-A06-p115948 | A06 | 521468 | 1.48E-05 |
|  | Bn-A06-p577934 | A06 | 681359 | 2.63E-05 |
|  | Bn-A06-p23666378 | A06 | 23268888 | 2.04E-05 |
| *qSRI.A07* | Bn-A07-p7392457 | A07 | 9202962 | 1.02E-06 |
|  | Bn-A07-p6437343 | A07 | 8247948 | 1.45E-06 |
|  | Bn-A07-p6824682 | A07 | 8635237 | 2.69E-06 |
|  | Bn-A07-p6541352 | A07 | 8351957 | 5.04E-06 |
|  | Bn-A07-p6373884 | A07 | 8184529 | 6.90E-06 |
|  | Bn-A07-p6542812 | A07 | 8353370 | 1.03E-05 |
|  | Bn-A07-p7392454 | A07 | 9202959 | 1.07E-05 |
|  | Bn-A07-p6552161 | A07 | 8362808 | 1.09E-05 |
|  | Bn-A10-p12485323 | A07 | 2519998 | 1.13E-05 |
|  | Bn-A07-p6553314 | A07 | 8363959 | 1.43E-05 |
|  | Bn-A07-p9987682 | A07 | 11798228 | 1.58E-05 |
|  | Bn-A07-p6309969 | A07 | 8120524 | 1.62E-05 |
|  | Bn-A07-p6534557 | A07 | 8345112 | 1.64E-05 |
|  | Bn-A07-p6553663 | A07 | 8364308 | 1.69E-05 |
|  | Bn-A07-p6553774 | A07 | 8364419 | 1.69E-05 |
|  | Bn-A07-p6554430 | A07 | 8364985 | 1.69E-05 |
|  | Bn-A07-p6576078 | A07 | 8386744 | 1.69E-05 |
|  | Bn-A07-p6576689 | A07 | 8387294 | 1.69E-05 |
|  | Bn-A07-p6544677 | A07 | 8355323 | 1.85E-05 |
|  | Bn-A07-p6327847 | A07 | 8138402 | 1.87E-05 |
|  | Bn-A07-p6575320 | A07 | 8385965 | 2.07E-05 |
|  | Bn-A07-p7390413 | A07 | 9200958 | 2.32E-05 |
|  | Bn-A07-p6554767 | A07 | 8365412 | 2.43E-05 |
|  | Bn-A07-p9441979 | A07 | 11252484 | 2.48E-05 |
| *qSRI.A09* | Bn-A09-p30171993 | A09 | 34170386 | 4.44E-09 |
|  | Bn-A09-p30592138 | A09 | 34590741 | 4.46E-07 |
|  | Bn-A09-p30010889 | A09 | 34009492 | 5.23E-07 |
|  | Bn-A09-p28910059 | A09 | 32908662 | 1.09E-05 |
|  | Bn-A09-p28914189 | A09 | 32912541 | 1.09E-05 |
|  | Bn-A09-p29756068 | A09 | 33754463 | 2.88E-05 |
| *qSRI.C02* | Bn-scaff_15712_6-p214229 | C02 | 40565480 | 6.49E-06 |
| *qSRI.C05* | Bn-scaff_17869_1-p1058624 | C05 | 19589640 | 1.14E-06 |
|  | Bn-scaff_17869_1-p1067608 | C05 | 19600357 | 2.17E-06 |
|  | Bn-scaff_21338_1-p1085673 | C05 | 16613154 | 5.36E-06 |
